# Motor activities to improve maths performance in pre-school children with typical development

**DOI:** 10.3389/fpsyg.2024.1332741

**Published:** 2024-05-22

**Authors:** Pedro Flores, Eduarda Coelho, Maria Isabel Mourão-Carvalhal, Pedro Forte

**Affiliations:** ^1^Department of Sports, Higher Institute of Education and Sciences of the Douro, Penafiel, Portugal; ^2^Department of Sports, University of Trás-os-Montes and Alto Douro, Vila Real, Portugal; ^3^Research Center in Sports, Health and Human Development, Covilhã, Portugal; ^4^Research Center for Active Living and Well Being (Livewell), Instituto Politécnico de Bragança, Bragança, Portugal

**Keywords:** pre-school, mathematical skills, visuomotor integration skills, spatial skills, gross motor skills

## Abstract

Poor maths skills are associated with negative outcomes throughout life, such as lower academic qualifications, decreased professional success and socio-economic results. Mathematical skills emerge continuously throughout childhood and those that children acquire in pre-school are crucial for activities that support analytical thinking, problem-solving and reasoning and argumentation skills. Many of these activities are related to motor skills, since certain cognitive and motor areas of the brain are activated simultaneously when solving maths problems. Of all motor skills, visuomotor integration skills have been documented as those that are most consistently positively and significantly associated with maths performance in pre-school children. These skills are influenced by visual perception (spatial and attention skills), fine motor coordination and gross motor skills. Early intervention can improve visuomotor integration skills in pre-school children. Of all skills that make up visuomotor integration, spatial skills, in addition to being the first skills to influence numerical knowledge and the recognition of geometric shapes, are also those skills that form part of the majority of programs and activities to be worked on with pre-school children for the development of mathematical concepts. However, most intervention programs or activities to develop spatial skills are carried out in the classroom, usually through activities involving handling small objects. In this sense and given the significant association between visuomotor integration skills and gross motor skills, the main objective of this study was to list a set of activities to develop spatial skills, with a strong involvement of gross motor skills, in a classroom, playground or home context.

## 1 Introduction

Mathematics is a way of thinking about the world and organizing experiences, involving reasoning and problem solving (Spodek, 2002). Poor maths skills are associated with negative outcomes throughout life, such as lower academic qualifications (Duncan et al., 2007; Chernyak et al., 2016), decreased professional success (Parsons and Bynner, 2005) and socio-economic outcomes (Ritchie and Bates, 2013). The development of these skills occurs in a hierarchical way (Von Aster and Shalev, 2007), already present in babies through discrimination of the numerosity of two sets (Hyde, 2011; Starr et al., 2013) and sense of measure (greater than and more than…) (Geist, 2009). In this sense, maths is learnt before school through numbers and quantities (McWayne et al., 2004; Geist, 2009). However, mathematical skills emerge continuously throughout childhood (Geist, 2009) and those that children acquire in pre-school are crucial for activities that support analytical thinking, problem solving and reasoning and argumentation skills (Clements et al., 2004). In this sense, it is essential in pre-school education to give continuity to this learning, which requires experiences related to their interests in everyday life, when they play and explore their daily lives (Silva et al., 2016), since at this stage of education children enjoy activities that develop their mathematical skills (Ginsburg et al., 2006).

Many of these activities are related to those that require bodily movement, and according to the theory of “*Embodied Cognition*,” cognition emerges from the individual’s “*coupling*” (embodied relationship) with the physical and social context, as a result of sensorimotor activity (Smith, 2005; Wilson and Foglia, 2011; Soylu and Newman, 2016). According to this theory, mathematical skills are interconnected with motor skills, since representations of distance, quantities and numbering are based on bodily experiences (Link et al., 2013; Fisher et al., 2018) and certain cognitive and motor areas of the brain are activated simultaneously when solving mathematical problems (Fischer and Brugger, 2011). Also, the idea of “learning to learn” suggests that early learning is centered around the motor system and as the child adapts to changes, cognitive and motor skills develop simultaneously (Adolph, 2005). Today, there is neurophysiological and neuroimaging evidence that the prefrontal cortex, cerebellum and connecting structures are coactivated in certain cognitive and motor tasks, suggesting an interrelationship between motor and cognitive development (Diamond, 2000; Abe and Hanakawa, 2009). In this sense, motor skills influence academic performance in the early years (Alvarez-Bueno et al., 2017;; Macdonald et al., 2018; De Waal, 2019; Duncan et al., 2019; Malambo et al., 2022), being described as one of the criteria for school readiness (Department for Education, 2020; Jones et al., 2021).

### 1.1 Maths curriculum guidelines for pre-school

Since the development of mathematical notions begins at a very early age (McWayne et al., 2004; Geist, 2009; Hyde, 2011; Starr et al., 2013), it is essential to continue this learning in pre-school (Silva et al., 2016), as the knowledge acquired in the early years will positively influence later learning (Clements et al., 2004).

Learning maths at these ages should be centered on activities that are meaningful to the child and that are associated with other content areas (Silva et al., 2016). Thus, according to the “Curriculum Guidelines for Pre-School” (Silva et al., 2016), in order to develop the various mathematical notions, the educator must take into account: (1) General processes; (2) Mathematical components.

**Figure 1:**
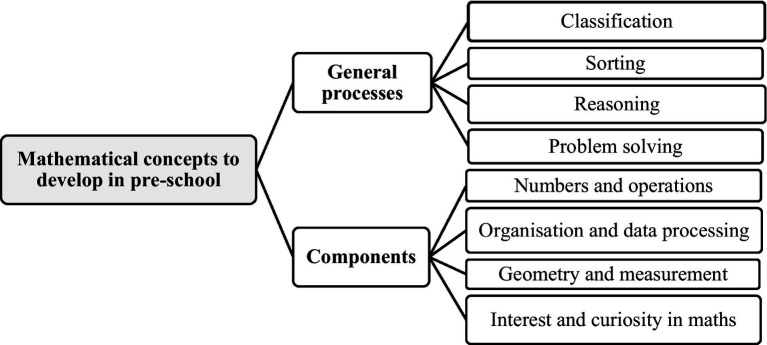
Mathematical notions to be developed according to the curriculum guidelines for pre-school (Silva et al., 2016).

The general processes are a set of processes that are transversal to the approach to mathematics, namely classification, sorting, reasoning and problem solving, and the mathematical components concern numbers and operations, organization and data processing, geometry and measurement, and interest and curiosity in mathematics (Figure 1).

#### 1.1.1 General processes

*Classification* implies that the child is able to distinguish, organize and establish relationships between objects by equality or difference.

*Sorting* implies that the child is able to order objects by quantity, height, size, thickness, speed and duration.

*Mathematical reasoning* involves using objects where children are encouraged to explain and justify solutions. Recognizing, understanding, and creating sequences of patterns are important elements in the development of mathematical reasoning.

*Problem solving* is the process of appropriating and integrating mathematical learning. The problems proposed to the child must have meaning for them (everyday activities) and the educator must use games and play for this purpose.

Regarding the mathematical components, four approaches are proposed: numbers and operations, data organization and processing, geometry and measurement, and interest and curiosity in mathematics.

#### 1.1.2 Maths components

##### 1.1.2.1 Numbers and operations

Numbers are abstractions that apply to a wide range of real and imaginary situations. They do not exist in isolation but make up a system of relationships and operations by which they can be compared, added, subtracted, multiplied and divided. It is these relationships that apply to a wide variety of problems (National Research Council, 2009). The development of number sense is progressive, as counting involves knowing the number sequence and matching term to term (Wynn, 1992; Sarnecka and Carey, 2008). Also, the ordering of numerals and the ability to compare magnitudes is related to the construction of a mental number line, where children become aware of the relationship between numbers (5 is more than 4; 6 is more than 5) (Silva et al., 2016).

Operations refer to basic arithmetic skills such as adding and subtracting and are used to relate quantities. Children are only prepared to develop these skills when they understand the concepts of cardinality and counting. These skills prepare children to develop more complex arithmetic skills such as multiplication and division (Barth et al., 2008; Canobi and Bethune, 2008).

#### 1.1.3 Organization and data processing

The collection, organization and data processing is based on classification, counting and comparison. Statistics, as the quantitative analysis of data, is a very important area of maths that provides multiple opportunities for numerical development. In kindergarten life, there are many opportunities to collect, organize and interpret quantitative data from everyday situations and from carrying out experiments and projects (Silva et al., 2016).

#### 1.1.4 Geometria e medida

Geometry and measurement provide systems for describing, representing, and understanding the world. Geometry is the study of shapes and spaces (two-dimensional—2-D and three-dimensional—3-D). Measurement has to do with how to determine the size of object shapes (National Research Council, 2009). In everyday life, these skills are present in countless situations and can be mobilized so that the child realizes how useful they are in everyday life (Silva et al., 2016).

#### 1.1.5 Geometry

Strongly associated with spatial development (orientation and spatial visualization) (Gelman and Williams, 1997) and analysis and operations with shapes (Anderson, 2000).

Spatial development includes two main skills, spatial orientation, and spatial visualization of images. Spatial orientation involves knowing where you are and how to get around in the world (Gelman and Williams, 1997). Children learn words like “next to” and “between.” Later, they learn words referring to frames of reference, such as “in front of,” “behind.” The words “left” and “right” are learnt much later, and are a source of confusion for several years (Gopnik and Meltzoff, 1986). In these early years, children can also learn to analyze a route through a space (Wang and Spelke, 2002). A visualização espacial de imagens é compreender e executar movimentos imaginados de objetos 2-D e 3-D. Para o efeito, é necessário ser capaz de criar uma imagem mental e manipulá-la através de uma estreita relação entre estas duas capacidades cognitivas. A visualização espacial de imagens tem sido positivamente associada à construção e composição de formas (Sarama et al., 1996).

Analysis and operations with shapes is the basic way in which children learn the names of objects (Jones and Smith, 2002) and the ability to recognize and combine shapes (Anderson, 2000).

In this sense, it is through spatial development, as well as the relationship and manipulation of objects, that children can learn what is “far” and “near,” “inside,” “outside” and “between,” “open” and “closed,” “above” and “below,” which also allows them to recognize and represent different geometric shapes that they will gradually learn to differentiate, name and characterize (Silva et al., 2016).

#### 1.1.6 Measurement

Measuring is a process that involves children starting to identify the measurable attributes of objects (length, weight, capacity, volume, time, temperature, etc.) from their everyday experiences (Silva et al., 2016). Initially, this process is based on directly comparing and ordering objects (longer, shorter, of equal length, heavier, lighter, etc.), gradually making it more difficult by using non-standardized units of measurement (cup, foot or shoe, etc.). These experiences enable children to gradually understand the usefulness of measuring instruments and standardized measures, as these are also part of their daily lives (Silva et al., 2016).

One way of more formally assessing children’s understanding of measurement is through comparison tasks (Mullet and Paques, 1991).

#### 1.1.7 Interest and curiosity in maths

Maths is present in the majority of children’s everyday activities (National Research Council, 2009). In this sense, the teacher has a fundamental role to play in developing their interest and curiosity by drawing the child’s attention to the presence of maths in the world around them (Silva et al., 2016). In this sense, the aim is to stimulate children’s natural curiosity by providing them with favorable mathematical experiences so that mathematics becomes an engaging and permanently interesting challenge (Geist, 2009).

### 1.2 Motor skills

Traditionally, motor skills are divided into two categories, gross motor skills (GMS) and fine motor skills (FMS) (Grissmer et al., 2010; Oberer et al., 2017; Goodway et al., 2019). As for GMS, they basically use movements produced by large muscle groups. They include locomotor skills, which involve moving the body in space (walking, running, jumping and sliding), postural or balance skills, which refer to the ability to maintain a controlled position or posture during a task (dynamic balance—maintaining position in activities that require movement; or static balance—maintaining position in stationary tasks) and manipulative skills, used to control objects in actions with the hands or feet (grasping, tapping, absorbing, lifting, etc.)., which can be propulsive (sending objects) or receptive (receiving objects) (Lopes et al., 2013; Magistro et al., 2015; Kokstejn et al., 2017; Haywood and Getchell, 2019). FMS are defined as movements produced by small muscle groups. One type of FMS is fine motor coordination (FMC) or visuomotor coordination, which refers to movements involving hand-eye coordination, manual dexterity, motor sequencing and speed and precision, and can also be referred to as non-graphomotor skills (Davis and Matthews, 2010; Suggate et al., 2018). Another type of FMS is visual and motor integration, called visuomotor integration (VMI) or visuospatial integration, which refers to the organization of small muscle movements of the hand and fingers through the processing of visual and spatial stimuli, based more on synchronized hand-eye movements (Carlson et al., 2013; Goodway et al., 2019) and are typically tasks involving writing, drawing, copying shapes, letters or other stimuli (Beery and Buktenica, 1997; Oberer et al., 2017), which can be referred to as graphomotor skills (Davis and Matthews, 2010).

**Figure 2:**
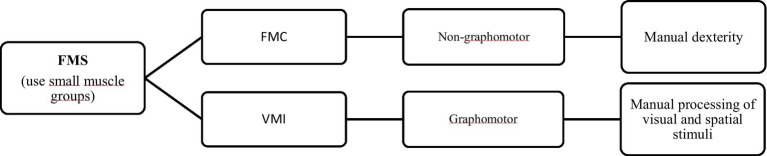
Summary of fine motor skills categories (adapted from Flores et al., 2023a).

Among these skills, recent systematic review studies concluded that it was mainly the FMS that justified mathematical academic performance in preschool children (van der Fels et al., 2015;Macdonald et al., 2018; Flores et al., 2023a; Figure 2).

Although a relationship between VMI and FMC has been demonstrated (Sortor and Kulp, 2003; Beery and Beery, 2006; Carlson et al., 2013; Byers et al., 2016), where children with better FMC may be better at manipulating objects, which allows them to direct additional attention resources to new learning, namely VMI (Kim et al., 2018), FMC has not been found to be a consistent predictor of later academic performance (Kim et al., 2018). In this sense, among the FMS, VMI skills have been documented as those that are most consistently positively and significantly associated with maths performance (Macdonald et al., 2018; Flores et al., 2023a).

### 1.3 Development of the VMI and its connection to maths

The development of VMI skills is very sensitive and dynamic with rapid growth between the ages of 4 and 7 (Decker et al., 2011), peaking between the ages of 4 and 5 (Fang et al., 2017), but continuing until at least the age of 12 (Decker et al., 2011). In this sense, considering the age of rapid growth of the VMI and the age of preschool attendance, it is important that this skill is well worked on at this academic stage.

**Figure 3:**
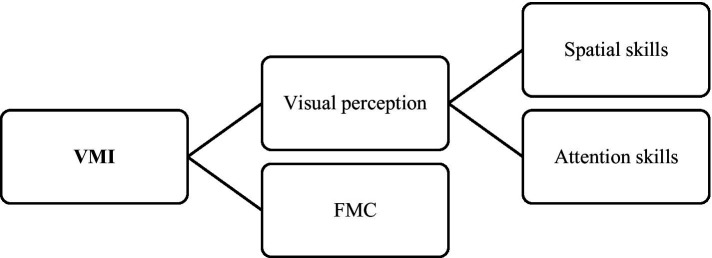
Components of VMI.

The literature has been consistent in describing that VMI skills are a multidimensional construct and its main components are visual perception and FMC skills (Osborn et al., 1984; Korkman et al., 1998; Tseng and Chow, 2000; Beery and Beery, 2006, 2010; Newcombe and Frick, 2010; Carlson et al., 2013; Dinehart and Manfra, 2013; Memisevic and Hadzic, 2013; Wang et al., 2013; Verdine et al., 2014; Goodway et al., 2019). VMI is defined as a person’s ability to visually perceive and understand the spatial relationships between objects and to manipulate, construct or reproduce models using the FMC (Korkman et al., 1998; Beery and Beery, 2010; Carlson et al., 2013; Verdine et al., 2014). However, success in tasks that require VMI skills not only requires children to coordinate their visual, spatial and motor skills (Verdine et al., 2014), but also attention control (Korkman et al., 1998; Beery and Beery, 2004; Becker et al., 2014). In this sense, VMI requires the integration of visual and spatial skills and executive attention, as well as FMC (Beery and Beery, 2004; Figure 3).

Vision is the system best equipped to structure space, since it structures, organizes and interprets all the spatial dimensions, and only then can motor actions come into play. It is between the ages of 3 and 7 that children should master all the notions of orientation: up, down, in front, behind, inside/outside, big, small, high, low, here, there, near, far, etc. In this sense, if children have spatial difficulties, they may have problems with localization, orientation, conservation of distance, surface area, volume and speed, which are the basis for the formulation of many mathematical concepts (Fonseca, 2010).

It has been documented that tasks requiring VMI skills, i.e., the integration of motor and visual processes, are highly related to the development of mathematical skills (Zebian, 2005; Puranik and Lonigan, 2012; Becker et al., 2014). This skill allows children to represent quantities, reproduce and perform transformations of shapes (Cameron et al., 2019). These activities are strongly associated with the development of quantitative tasks, numerical representations and mathematical performance (Verdine et al., 2017).

The literature has been consistent in supporting that in preschoolers executive function and VMI contribute unique and shared variance to mathematics performance (Cameron et al., 2012; Becker et al., 2014; Cragg and Gilmore, 2014; Verdine et al., 2014), revealing bidirectional influences between mathematics, executive function and VMI (Brock et al., 2018a). Executive function refers to higher-order cognitive skills used in information processing and problem solving (Blair, 2010; Beck et al., 2011) and its components include inhibition, cognitive flexibility, working memory, planning and updating (Blair and Razza, 2007; Herbers et al., 2011).

In early childhood, executive function and VMI co-develop (Cameron et al., 2016). Although these skills are considered separate processes, any task that requires visual perception (spatial and attention skills) to solve problems will require executive function (Korkman et al., 1998). However, it has been suggested that VMI is related to mathematical performance even after executive function has been controlled, specifically in geometry (spatial reasoning) (Duran et al., 2018). Verdine et al. (2014) concluded that even when the effects of other variables are removed, such as vocabulary and executive function, spatial abilities are a unique predictor of overall math performance, explaining 27.1% of its variability. In this sense, VMI skills can independently contribute to math performance (Verdine et al., 2014; Duran et al., 2018).

Also, in a recent study by Flores et al. (2023b), the authors concluded that VMI directly, positively and significantly influenced maths performance in preschool children with typical development and that GMS could indirectly contribute to maths performance, justified by the positive and direct associations with VMI skills. In this sense, the results of this study suggest the inclusion of GMS in structured programs to develop VMI and thus contribute to mathematical performance (Flores et al., 2023b).

Although deficits in any of these processes affect the results of VMI (Daly et al., 2003; Carlson et al., 2013; Van Wyk et al., 2020), all these skills are malleable and can be trained (Diamond and Lee, 2011; Uttal et al., 2013).

VMI skills are used in most everyday tasks with an emphasis on early maths learning (Mix and Cheng, 2012; Verdine et al., 2014; Zhang and Lin, 2015; Mix et al., 2016). It has been shown that basic maths skills are directly dependent on VMI (Cameron et al., 2012; Becker et al., 2014; Carlson et al., 2014; Grissmer et al., 2014; Verdine et al., 2014), which is justified by the fact that classroom tasks often require this type of skill (Dehaene and Cohen, 2007). In addition to VMI skills influencing maths skills in preschool, they are a significant predictor of maths when children enter school (Gunderson et al., 2012; Rittle-Johnson et al., 2019). Furthermore, research in cognitive neuroscience points to a neural basis that links VMI skills with maths (Hubbard et al., 2005; St Clair-Thompson and Gathercole, 2006; Dehaene, 2011), namely in numerical operations skills (Dehaene et al., 2004; Hubbard et al., 2009) and damage to the parietal cortex often results in deficiencies in spatial and numerical skills (Bueti and Walsh, 2009).

In a recent systematic review carried out by Flores et al. (2023a), the authors found that all the mathematical skills proposed by National Research Council (2009) for preschool were associated with VMI skills: numeracy (Verdine et al., 2014; Osorio-Valencia et al., 2017; Duran et al., 2018; Kim et al., 2018; Nesbitt et al., 2019; Escolano-Pérez et al., 2020; Khng and Ng, 2021), addition and subtraction (Becker et al., 2014; Brock et al., 2018a; Duran et al., 2018; Cameron et al., 2019; Nesbitt et al., 2019; Khng and Ng, 2021), measurement (Dinehart and Manfra, 2013; Manfra et al., 2017; Duran et al., 2018; Kim et al., 2018; Nesbitt et al., 2019; Greenburg et al., 2020) and geometry (Manfra et al., 2017; Duran et al., 2018; Kim et al., 2018; Greenburg et al., 2020).

Since the development of VMI skills seems to be positively influenced by the visual perception skills (spatial and attention skills) of FMC and GMS, it was necessary to understand the relationship between these components of VMI and maths skills.

#### 1.3.1 Spatial skills and its connection to maths

Spatial ability is a generic term for a multitude of related abilities that include the mental manipulation of information about objects in the environment and spaces we inhabit (Uttal et al., 2013). A recent meta-analysis sought to define spatial abilities and concluded that there were four distinct types of spatial abilities based on the intersection of two dimensions. One is related to information, which can be intrinsic and extrinsic, and the other is related to tasks, which can be static and dynamic (Uttal et al., 2013). Intrinsic information concerns the properties of an object (constitution/shape) and extrinsic information concerns the location of the object (left/right/front/back/near/far, etc.). In terms of tasks, static tasks do not change the object’s properties (shape or position), while dynamic tasks change the object’s properties. Spatial skills develop very early in children through everyday activities, such as observing and exploring their surroundings (Barros and Palhares, 2001; Uttal et al., 2013). With regard to tasks, in static tasks the object’s properties (shape or position) do not change, while in dynamic tasks the object’s properties change. Spatial skills are developed very early in children through everyday activities such as observing and exploring their surroundings (Barros and Palhares, 2001; Uttal et al., 2013). These activities allow children to explore space and become aware of their relationship and position with objects (near/far/outside/inside/open/closed/top/bottom), as well as progressively recognizing their geometric shapes (Silva et al., 2016). There is a growing realization that spatial abilities are an important aspect of intellectual capacity and that they are separable from general intelligence (Hegarty and Waller, 2004), and although they are always present in children’s daily lives, they have been largely ignored in formal educational environments (Clements and Sarama, 2011). However, some efforts have already been made to include this approach in school environments (Moss et al., 2015; Silva et al., 2016).

Literature has shown a strong association between these spatial and mathematical skills (Ansari et al., 2003; Gunderson et al., 2012; Mix and Cheng, 2012). A factor analysis found that these skills are significant predictors of maths skills in preschool, third and sixth grades (Mix et al., 2016). In children aged between 4.5 and 7.5 years, positive and significant relationships have been identified in tasks that required children to remember and reproduce a series of spatial locations with number naming and number magnitude processing (LeFevre et al., 2010), as well as problem solving (Cheng and Mix, 2014; Zhang and Lin, 2015; Skagerlund and Träff, 2016). Children who develop better spatial representations acquire additional mathematical skills earlier (Bachot et al., 2005; Bailey et al., 2014). In a study carried out by Hawes et al. (2017), which aimed to investigate the extent to which spatial learning supports children’s numerical development, the results showed that only those in the intervention group made significant gains in mathematical numerical comparison skills. These skills are strongly related to simultaneous and subsequent mathematical performance (De Smedt et al., 2013; Nosworthy et al., 2013).

There is evidence that early spatial skills longitudinally predicted early numerical knowledge skills (such as plus, minus, equals and second) and that spatial skills assessed at 3 years and later at 4 years, even after controlling for executive functioning and vocabulary skills, predicted approximately 15% of the variability in early number knowledge (Verdine et al., 2014), thus early interventions aimed at improving spatial skills are justified (De Smedt et al., 2013; Grissmer et al., 2013).

Mathematical concepts are also based on mental representations of objects that are developed by the child’s interaction with physical objects through play and games (Ginsburg, 1977; Clements and Sarama, 2011) where children are able to represent and interpret numerical information spatially (Gunderson et al., 2012) and use strategies to solve problems (Mata et al., 2011). Recently, it has been suggested that infants’ spatial processing acts as a later precursor to maths at the age of 4 (Lauer and Lourenco, 2016). In addition, spatial memory in young children has been associated with their mathematical performance (Holmes et al., 2008; LeFevre et al., 2010; Meyer et al., 2010; Hornung et al., 2011; Passolunghi and Mammarella, 2012; Szucs et al., 2013). Given the importance of spatial memory, it has been recommended that it should be given the same importance and the same amount of instructional time as numeracy from pre-school to 8th grade (National Council of Teachers of Mathematics, 2006). However, geometry and spatial thinking are often minimized in pre-school education (Clements and Sarama, 2011). This can be explained by the fact that preschool teachers receive little professional training in geometry and spatial thinking (Ginsburg et al., 2006), compared to other maths topics (Lee, 2010). During preschool education, it is essential that children are able to use symmetry, create mental images of geometric shapes, recognize and represent shapes from different perspectives and assemble and disassemble two- and three-dimensional shapes (Frick et al., 2014; Hawes et al., 2015). In this sense, there is an urgent need for more training for preschool teachers on the importance and teaching of spatial skills in pre-school education. It has been shown that children show high levels of motivation during activities involving spatial skills (Naqvi et al., 2013; Taylor and Hutton, 2013).

#### 1.3.2 Executive attention skills and its connections to maths

Regarding attention, theoretical and empirical evidence, supported by the results of neuroimaging studies, indicates that the processes of attention and VMI are related (Diamond, 2000; Floyer-Lea and Matthews, 2004), since tasks that require VMI also require attentional control (Beery and Beery, 2004; Becker et al., 2014). In the light of the embodied cognition theory, attention control and VMI co-develop as children interact with the environment (Campos et al., 2000). Also, automaticity theory argues that cognitive resources become available as someone is able to automatize or perform a task without using all their attention, which in turn makes it easier to simultaneously perform a second task that requires attention (Floyer-Lea and Matthews, 2004). In this sense, children who automate a task that requires VMI do not need to pay as much attention to the execution of visual and motor movements, so they can have additional cognitive resources available for other tasks (Floyer-Lea and Matthews, 2004). In this sense, executive attention is related to the development of a variety of early mathematical skills in the face of the influence exerted by VMI (LeFevre et al., 2013).

#### 1.3.3 FMC skills and its connection to maths

FMC refers to muscular coordination that produces minute and precise movements (Kimmel and Ratliff-Schaub, 2011) without strongly needing visual and spatial information (Korkman et al., 1998; Carlson et al., 2013). Although FMC does not depend on visual and spatial information, these motor skills can be relevant for many tasks in the early years of schooling (Marr et al., 2003). Since VMI involves the mental representation of an image that is replicated by controlling the minute movement of the fingers (Carlson et al., 2013), FMC plays a very important role in school success (Roebers et al., 2014; Kim et al., 2015; Fischer et al., 2020), since children with better FMC may be better at manipulating objects, such as pencils or notebooks, which allows them to direct additional attention resources to learning instead of focusing them on movements associated with FMC (Kim et al., 2018). In this sense, a child with good FMC, when performing an academic task, can impose a lower cognitive load compared to a child who still shows difficulties in FMC (Floyer-Lea and Matthews, 2004; Luo et al., 2007; Cameron et al., 2015). Therefore, FMC is considered a precursor to VMI (Kim et al., 2016) and the correlations are positive and significant (Carlson et al., 2013).

In a recent systematic review, one of the aims of which was to identify the specific motor skills that were positively associated with mathematics in pre-school children, the authors concluded that VMI was, among all the motor skills, the one that stood out the most (Flores et al., 2023a). However, the authors also identified positive and significant relationships between FMC and mathematical skills. A cross-sectional study involving 4- and 5-year-old preschool children concluded that performance on FMC significantly predicts fractional reasoning tasks (R2 = 0.258; *p* = 0.003) (Clark et al., 2021). Other cross-sectional studies found that FMC was related to finger-based numerical representations (Suggate et al., 2017; Fischer et al., 2020) and counting (Manfra et al., 2017; Fischer et al., 2018). Fingers have long been used to help with counting and calculation and their use in early counting is almost universal (Butterworth, 1999; Crollen and Noël, 2015). Using the finger not only helps children learn to count, but can also help them understand the meaning of numbers (Fischer, 2008; Fischer et al., 2018). Recent studies have found that only CMF (agility and dexterity) predicted initial calculation skills in the early years (Suggate et al., 2017; Fischer et al., 2018). However, as the child’s age progresses, finger dexterity no longer correlated significantly with ordinal and cardinal representations, and it is possible that the child’s spatial abilities play a more important role in counting (Fisher et al., 2020). Therefore, given the importance of manual dexterity in counting in the early preschool years, the use of the finger should be encouraged to develop numerical skills and simultaneously train these skills (Fisher et al., 2020). Longitudinal studies have also positively associated FMC with later mathematical performance (Dinehart and Manfra, 2013; Osorio-Valencia et al., 2017; Kim et al., 2018; Greenburg et al., 2020). In this sense, given the relationship between FMC and VMI and mathematical skills, these types of motor skills should be developed through intervention programs.

#### 1.3.4 GMS and its connections to maths

GMS refers to movements produced by large muscle groups (Haywood and Getchell, 2019) and there is neural evidence to support that the development of these motor skills stimulates the development of the central nervous system, contributing to the development of VMI (Mujkic and Papric, 2013; Wang et al., 2015; Fang et al., 2017; Zhang et al., 2019). It has also been argued that in the same motor action it is complex to clearly differentiate the independent involvement of each of the motor skills (GMS and FMS), since they coexist and are fundamental for the efficient performance of the task (Payne and Isaacs, 2012; Flatters et al., 2014). This can be justified by the fact that higher-order neuromotor processes seem to be involved simultaneously in GMS and FMS, since these processes do not occur independently (Roebers and Kauer, 2009; Oberer et al., 2017). Thus, children with difficulties in GMS are more likely to show problems in VMI skills (Wassenberg et al., 2005; Oberer et al., 2017).

Although some authors suggest significant associations between GMS and maths mastery (Son and Meisels, 2006; Pagani et al., 2010; De Waal, 2019), the literature is inconsistent and insufficient to report the relationships between specific components of GMS and maths performance in preschool (Macdonald et al., 2018; Escolano-Pérez et al., 2020; Macdonald et al., 2020; Flores et al., 2023a). Despite the inconsistency of the results, these skills should be part of the work of early childhood teachers, since this educational period should contribute to the integral development of children (Escolano-Pérez et al., 2020). In any case, this inclusion is justified by the fact that studies have concluded that GMS promote the development of social skills, physical well-being (Cameron et al., 2016; Goodway et al., 2019; Haywood and Getchell, 2019) and perceived athletic competence in childhood (Piek et al., 2006). In addition, preschool children involved in a structured, cognitively challenging GMS program could contribute to the improvement of mathematical skills through the direct effect exerted on the improvement of FMS (Hudson et al., 2021). Flores et al. (2023b), in a study carried out with pre-school children (between 3 and 6 years old), concluded that GMS could indirectly contribute to mathematical performance, justified by the significant, positive and direct associations with VMI skills. In this sense, the results of this study suggest the inclusion of GMS in structured programs to develop VMI and thus contribute to mathematical performance (Flores et al., 2023b).

As the literature shows, in order to promote the development of VMI skills, it will be necessary to include work on the visual perception skills (spatial and attention skills) of FMC and GMS in intervention programs.

## 2 Intervention programs to develop VMI

As already mentioned, all the processes that can directly influence VMI, namely visual perception (spatial skills and attention skills), FMC and GMS, are malleable and trainable (Diamond and Lee, 2011; Uttal et al., 2013). Early intervention improves VMI skills in preschool and early school-age children (Parush and Hahn-Markowitz, 1997; Dankert et al., 2003). There is evidence from the fields of physiotherapy and occupational therapy to indicate that early deficits in fine motor skills can be improved or corrected through interventions (Bayona et al., 2006; Ratzon et al., 2007). Most studies have proven the effectiveness of long interventions, at least 7 months, to improve VMI skills (Parush and Hahn-Markowitz, 1997; Case-Smith, 2002), however, a 3-month period of just 12 therapeutic sessions is of great importance for improving VMI (Ratzon et al., 2009).

In this sense, given the implications of VMI for mathematical skills, it is essential to include programs in the preschool curriculum that aim to explicitly train VMI skills (Uttal and Cohen, 2012; Newcombe et al., 2013; Brock et al., 2018b).

The literature has highlighted some programs to improve mathematical skills with the inclusion of activities that promote the development of VMI. Table 1 shows some of these programs, the objectives to be worked on related to VMI skills, the associated activities, the context in which the program is developed, and the main motor skills involved.

**Table 1:** Programs to develop VMI.

Author	Program	Main objective	Activities	Context	Motor skills to develop
Brock et al. (2018b)	*Minds in motion*	Developing spatial and FMC skills	Handling and guiding objects through specific spaces	Classroom	FMS
Sarama and Clements (2004)	*Building blocks*	Developing spatial skills	Create geometric shapes with Legos.	Classroom	FMS
Klein and Starkey (2002)	*Pré-K mathematics curriculum*	Developing spatial and FMC skills	Manipulating objects to reproduce shapes and patterns.	Classroom	FMS
Casey (2004)	*Storytelling*	Developing spatial and FMC skills	Oral storytelling using manipulative activities.	Classroom	FMS
Hohmann and Weikart (2002)	*High/Scope curriculum*	Developing spatial skills	Activities that work on shapes and patterns.	Classroom	FMS
Greenes et al. (2004)	*Big math for little kids*	Developing spatial skills	Identifying positions in space, orientating oneself and representing space using maps.	Classroom	FMS
Groth-Marnat and Teal (2000)	*Block design test*	Developing spatial skills	Reproducing patterns using small blocks.	Classroom	FMS
Moss et al. (2016)	*Math for young children (M4YC)*	Developing spatial skills	Standardization and mapping activities.	Classroom	FMS

Looking at Table 1, all the programs aim to develop VMI by working on spatial skills using manipulative objects to reproduce/construct geometric shapes (Hohmann and Weikart, 2002; Klein and Starkey, 2002; Casey, 2004; Sarama and Clements, 2004; Casey et al., 2008), patterns (Groth-Marnat and Teal, 2000; Hohmann and Weikart, 2002; Klein and Starkey, 2002; Moss et al., 2016; Brock et al., 2018b), positions and orientations in space (Hohmann and Weikart, 2002; Greenes et al., 2004; Moss et al., 2016; Brock et al., 2018b). Some of the activities that prevail in these programs are: matching games; manipulating and building with Legos and blocks (horizontally and vertically); constructing puzzles; going through and tracing labyrinths; making threads with various sequences (different patterns - size, colors, shapes, etc.); copying sequences of shapes and figures; making graphs; manipulating different objects to reproduce shapes and patterns; identifying positions in space.

A common element of all these intervention programs/activities to develop VMI is that they are applied in a classroom context without heavy reliance on GMS. In this sense, the aim of this article is to present and justify a set of activities to develop spatial skills with a significant involvement of GMS, to be applied in a classroom, playground or family (home) context.

## 3 Activities to develop spatial skills with a strong contribution from GMS

Spatial skills can be developed through specific physical activity programs for pre-school children (Hraste et al., 2018). For example, in Sweden, maths teaching in preschool is supported by physical activity and music (National Research Council, 2009). Elofsson et al. (2018) showed that children with and without motor skills problems benefited in mathematical learning when they were placed in an environment characterized by physical activity and music. These results are consistent with previous research (Donelly and Lambourne, 2011; Rasberry et al., 2011).

Structured physical activity programs for preschool children should focus on playful activities in the form of games (Geist, 2009; Yu et al., 2018; Zosh et al., 2018). These types of activities do not guarantee mathematical development, but they offer great opportunities for discovering mathematical concepts (Geist, 2009). Simple motor actions, exploring and mastering certain materials (balls, ropes, hoops, balloons, rackets, etc.), allow children to explore the relationship between their bodies and objects moving in space (Silva et al., 2016). There is evidence that using words such as inside, outside, below, above, near, far, etc. helps children to solve spatial problems and consequently improve their skills in this area (Pruden et al., 2011). However, some specific motor skills of locomotion or object manipulation are not acquired innately as children grow (Clark, 2005), they must be learned and practiced (Pic et al., 2018, 2020) through structured programs during early childhood (Logan et al., 2012; Robinson et al., 2015; Coutinho et al., 2016; Dapp et al., 2021), suggesting that early intervention could reduce or prevent potential mathematical difficulties (Duncan et al., 2007).

In this sense, there are manuals and books that promote mathematical performance through the exploration of motor skills. An extraordinary example is the book “Mathekings” (Hoenisch and Niggemeyer, 2007). According to the authors, children explore the world and discover mathematics through their senses. This book, designed for children aged between 4 and 8, allows them to work with and develop mathematical concepts such as quantities, sorting and matching, patterns and symmetry, numbers, geometry, space and time, weighing, measuring and comparing, graphs, matching and function, and even statistics, through the handling of everyday materials and body movements, whether in the classroom, on the playground or at home. Another example is the book Preschool Math (Williams et al., 2005). This book encourages educators to listen to and observe young children in order to better understand how they think about their world. The book uses these stimuli to develop useful and appropriate mathematical experiences in which children use their senses and bodies to explore ideas, record and talk about concepts and learn how mathematics is felt, tasted and seen. The proposed activities allow children to evaluate, explore, experiment, solve problems, make assumptions and form hypotheses, while using interesting materials and environments in a mathematical way. To enrich and diversify activities for children, the book “Why Play Matters: 101 Activities for Developmental Play to Support Young Children” (Essame, 2023), is full of play-based activities to support the development of children between the ages of 0 and 8. Based on the holistic and inclusive model of Developmental Play, which includes sensory play, creative-exploratory play, meaning-making play and higher play, the activities focus on supporting aspects of social, emotional, physical and cognitive development. Since play is fundamental to a child’s holistic development, this book is essential reading for early childhood professionals, elementary school teachers, occupational therapists and parents.

Spatial reasoning is an integral part of everyday life, and having good spatial skills strongly predicts children’s future performance in various subjects. In this sense, the books “Exploring the 3-D World: Developing Spatial and Math Skills for Young Children” (Hansel, 2021) and “Blocks and Beyond: Strengthening Early Math and Science Skills through Spatial Learning” are resources that promote the teaching of spatial skills in early childhood that contain research-based ideas and practical activities for early childhood educators to promote spatial development in children throughout the school day.

In this sense, considering the direct influence of GMS on VMI skills (Flores et al., 2023b) and that numerous programs to develop VMI skills include working on spatial skills through the manipulation of objects in a classroom context, the aim of this study was to present a set of activities to develop spatial skills, using playful activities with a strong involvement of GMS, which could be carried out in a classroom context, at the playground or at home.

The activities presented in the next section of this manuscript were taken and adapted from the book “Exercitar para Aprender” (Flores and Magalhães, 2019).

### 3.1 Ativities

All the proposed activities should be carried out in a playful way in the context of a game. The purpose of the activity should be well explained and practiced before the games begin. For each activity, it should be said: “let us see who does it well,” or “who is faster,” or “who hits the most,” etc. The teacher should correct whenever the child’s performance requires it (Weisberg and Zosh, 2018; Yu et al., 2018; Zosh et al., 2018). The proposed activities can be worked on in the classroom, playground or at home (Table 2).

**Table 2:** Activities to develop spatial skills with a strong contribution from GMS.

Objectives	N°	Description: ask the child to:
Directions (up, down, forward, backward, far, near, between, inside, outside, right, left, fast, slow)	1	Climb onto the chair (or other object) and stand on 2 feet.
	2	Move from one side of the chair to the other (or another object).
	3	Pick up an object and raise it above their head (choose other parts of the body or objects - above the knee, belly, etc.).
	4	Getting under a chair or table (or other objects).
	5	Placing an object below certain parts of the body (example: below the knees).
	7	Jumping together: first onto an object (you can use stair treads, Swedish benches, etc.) and then down.
	8	Jumping over a rope (gradually increasing the height).
	9	Jumping on your toes in the same place (first right foot, then left).
	10	Jumping on your toes around an object (e.g., a chair or table): first right foot, then left.
	11	Climbing stairs always using the right foot to start and then the left.
	12	Positioning yourself in relation to an object (e.g., chair): in front, behind, on the right side, on the left side, below and above.
	13	Two large squares (or other geometric figures) drawn on the board, spaced apart (they can be drawn on the playground floor, arches used, etc.): Throw a ball to hit: 1st into the square on the right; 2nd into the square on the left; 3rd above the right square; 4th, above the left square; 4th below the right square; 5th, below the left square; 6th between the squares; etc. (The squares can be drawn on the floor or arcs can be used).
	14	Place two objects (e.g., cones) approximately 1 m apart. The child stands approximately 3 m away and tries to pass the ball between the cones (score a goal). First right foot, then left foot.
	15	Throwing objects as far as possible (e.g., a ball): 1st with both hands behind the head; 2nd with the right hand; 3rd with the left hand.
	16	Throwing one object (e.g., a ball) closer to another (e.g., a target ball): 1st with both hands; 2nd with the right hand; 3rd with the left hand.
	17	Draw horizontal (then vertical) lines on the wall (or board) numbered 1–4. Throw a ball (the child should be close to the wall or board so that the probability of missing is minimal): above line 1; above line 2; below line 4; between line 2 and line 4; hit line 3; etc.
	18	The teacher rolls a ball across the floor and the child, sitting down, tries to hit the moving ball with another ball. First with the hand and then with the foot. (throw first slowly and gradually increase the speed of the throw).
Guidance	1	Carry out a short course drawn on the board or on a sheet of paper by the teacher.
	2	Treasure hunt: The teacher hides objects and shows them on a map where they have been hidden. The child tries to find the objects by following the directions on the map.
	3	The child makes a trajectory in relation to the surrounding space and then draws it on a sheet of paper.
	4	Playing “Blindfolded Goat” in a confined space. A blindfolded child tries to touch/catch one of the other children who is not blindfolded. The child who is caught swaps with the other.
	5	Draw a circuit in the classroom or playground and the blindfolded child must follow the route without touching the obstacles with the help of the teacher’s instructions (forward, stop, right, left, up, down, jump, lower, etc.).
	6	Set a distance (e.g., 5 m) and the child must adjust their movement to this distance: 1st, count the steps when walking normally; 2nd, take 1 step less; 3rd, 2 steps less; 4th, 2 steps more than when walking normally; 5th, jump together normally and count the number of jumps; 6th, 2 jumps less; 7th, 3 jumps less; etc.
Standardisztion	1	Beating with hands and feet: 3 claps +3 jumps; 2 claps +2 beats right leg +2 beats left leg; 2 claps +2 beats right leg +2 beats left leg +2 jumps; etc. (adjust the difficulty of the exercises to the children)
	2	Perform the following actions in succession: 2 giant steps +1 jump with your feet together; 2 giant steps +2 jumps with your feet together; 2 giant steps +3 jumps with your feet together; 3 giant steps +2 jumps with your feet together +2 hips; etc. (adjust the difficulty of the exercises to the children).
	3	Perform the following actions in succession (using a chair or other object): Climb the chair + go round the chair; climb the chair 2 times + go round the chair 1 time; climb the chair 1 time and go round the chair 2 times; climb the chair 1 time + go round the chair on toes + go round the chair on toes + go round the chair on toes; etc. (adjust the difficulty of the exercises to suit the children)
	4	Perform successive actions with the upper limbs: 3 lateral arm raises +3 anterior arm raises; 2 lateral arm raises +1 anterior arm raise; 3 lateral arm raises +2 anterior arm raises +2 lateral right arm raises +2 lateral left arm raises; etc. (adjust the difficulty of the exercises to the children)
	5	Perform successive actions with the lower limbs: Raise the right leg 3 times + raise the left leg 3 times; raise the right leg 2 times +1 time the left leg; raise the right leg 3 times +3 times the left leg + simultaneously move the legs apart and join 3 times. (Adjust the difficulty of the exercises to the children).
	6	Run or walk freely in a space and ask the children to form groups: Of 5, 4 or 3 elements; by color of shoes or clothes; etc.
	7	Perform a short aerobic dance routine with basic steps: 4 step touch +2 V-step +2 jumping Jack…. (gradually increase the difficulty according to the children’s characteristics).
Shapes/geometry	1	Running in a straight line; running with changes of direction to the right and left; always running in circular movements.
	2	Walking or running or jumping and executing the following geometric figures shown by the educator on the board or sheet of paper: Square; triangle; circle; rectangle, etc. (adjust the difficulty of the shapes to the children).
	3	The teacher draws the following geometric shapes in the space with their index finger: Square; triangle; circle; rectangle. The child must reproduce them while walking, running or jumping.
	4	Imitate the position and movement of certain animals: Dog; snake; frog; Kangaroo; etc.
	5	Form groups and ask them, hand in hand, to build shapes, figures, letters, numbers, etc. (if necessary, draw them on the board and show them): Circle; square; letter “L”; number “1”; etc.

## 4 Discussion

Given the importance of mathematics in future academic and professional outcomes (Parsons and Bynner, 2005; Duncan et al., 2007) there has been increasing attention to mathematical learning and development in preschool education (Cross et al., 2009; Elofsson et al., 2016). In this sense, it is important to give children the same opportunities to support early mathematical development through interventions (Jordan et al., 2012).

VMI skills have been documented as those that are most consistently positively and significantly associated with math performance in preschool children (Cameron et al., 2012; Carlson et al., 2014; Grissmer et al., 2014; Verdine et al., 2014; Flores et al., 2023a). These skills are influenced by visual perception (spatial and attention skills) (Ansari et al., 2003; Gunderson et al., 2012; Mix and Cheng, 2012; Mix et al., 2016), the FMC (Dinehart and Manfra, 2013; Manfra et al., 2017; Osorio-Valencia et al., 2017; Suggate et al., 2017; Fischer et al., 2018, 2020; Kim et al., 2018; Greenburg et al., 2020) and GMS skills (Son and Meisels, 2006; Pagani et al., 2010; De Waal, 2019). Of all the processes that influence VMI, spatial skills are worked on in most of the activities proposed to preschool children for the development of mathematical concepts. Although there are several educational programs aimed at developing arithmetic skills in preschoolers (Elofsson et al., 2018), these programs include working on spatial skills using board games (Elofsson et al., 2016). In this sense, most of these activities are carried out in the classroom context only through the handling of small objects, without strong dependence on GMS (Elofsson et al., 2018).

Given the significant association between GMS and VMI, the aim of this study was to propose a set of activities to develop spatial skills, with a strong participation of GMS in the classroom, playground or at home.

The inclusion of GMS is not only due to the fact that these skills contribute directly to the development of VMI skills, but is also justified by the fact that the main objective of this educational period is to contribute to the child’s overall development (Goodway et al., 2019; Haywood and Getchell, 2019). However, the reality is that the work on motor skills in preschool has not been highly valued, a fact that seems to be due to the common misconception that children develop their motor skills naturally (Escolano-Pérez et al., 2020). The truth is that the development of motor skills is related to practicing them (Logan et al., 2012; Pic et al., 2020). However, learning focuses primarily on academic content, limiting preschool children’s opportunities to develop motor skills in these educational environments (Cameron et al., 2016; Macdonald et al., 2020).

A meta-analysis of 50 studies showed that 25 of the studies reported a positive correlation between physical activity and math learning (Rasberry et al., 2011). Donelly and Lambourne (2011) revealed that students who integrated physical activity into their learning activities performed significantly better than the control group in mathematics. Studies have also shown that there is a correlation between children’s motor skills and mathematical performance (Lopes et al., 2013; Elofsson et al., 2018) and that children’s motor skills explained almost 16% of the variation in mathematical measures (Elofsson et al., 2018). Thus, a learning environment characterized by the inclusion of physical activity seems to have a positive effect on students’ general and specific learning of mathematics.

Structured physical activity should be a routine part of the preschool curriculum (Escolano-Pérez et al., 2020). These activities should be taught primarily in a playful way, as this is the most natural way for children to learn and develop (Yu et al., 2018; Zosh et al., 2018).

Educators often lament the lack of resources and materials available to implement practices that improve preschoolers’ motor skills (Robinson et al., 2012). In this sense, this article seeks to respond to the current need to present a set of practical motor activities that make it possible to work on spatial skills, with a strong participation of GMS and thus develop VMI skills and consequently mathematical skills.

It will therefore be necessary for educators to increase play opportunities through physical activity programs to develop spatial skills, with a strong participation of the GMS, to ensure the development of VMI skills and thus contribute to the development of mathematical skills.

## 5 Conclusion

Since motor skills are not acquired innately, they must be learnt and practiced. In this study, the proposal to include gross motor skills in activities to develop visuomotor integration skills, namely spatial skills, will not only contribute to mathematical learning, but also the possibility of developing other coordinative and conditional physical skills, and contribute to the child’s health. In this sense, it is up to the teacher, depending on the material and spatial conditions, to select a set of activities, adjusted to the characteristics and abilities of the children, to develop visuomotor integration skills, through spatial skills, with a large involvement of gross motor skills.

## Author contributions

PFl: Writing – original draft, Conceptualization. EC: Writing – review & editing, Supervision. MM-C: Writing – review & editing, Supervision. PFo: Writing – review & editing, Supervision.
